# 3-Cyclo­hexyl-2-thioxo-1,3-thia­zolidin-4-one

**DOI:** 10.1107/S1600536809045851

**Published:** 2009-11-07

**Authors:** Durre Shahwar, M. Nawaz Tahir, Asma Yasmeen, Naeem Ahmad, Muhammad Akmal Khan

**Affiliations:** a Department of Chemistry, Government College University, Lahore, Pakistan; b Department of Physics, University of Sargodha, Sargodha, Pakistan

## Abstract

In the title compound, C_9_H_13_NOS_2_, the complete mol­ecule is generated by crystallographic mirror symmetry, with all the non-H atoms of the rhodanine (2-thioxo-1,3-thia­zolidin-4-one) system and two C atoms of the cyclo­hexyl ring lying on the reflecting plane. The conformation is stabilized by intra­molecular C—H⋯O and C—H⋯S inter­actions. In the crystal, weak π–π inter­actions at a distance of 3.8140 (5) Å between the centroids of the heterocyclic rings occur.

## Related literature

For related structures, see: Shahwar *et al.* (2009*a* ▶,*b* ▶,*c* ▶,*d* ▶). For graph-set notation, see: Bernstein *et al.* (1995 ▶).
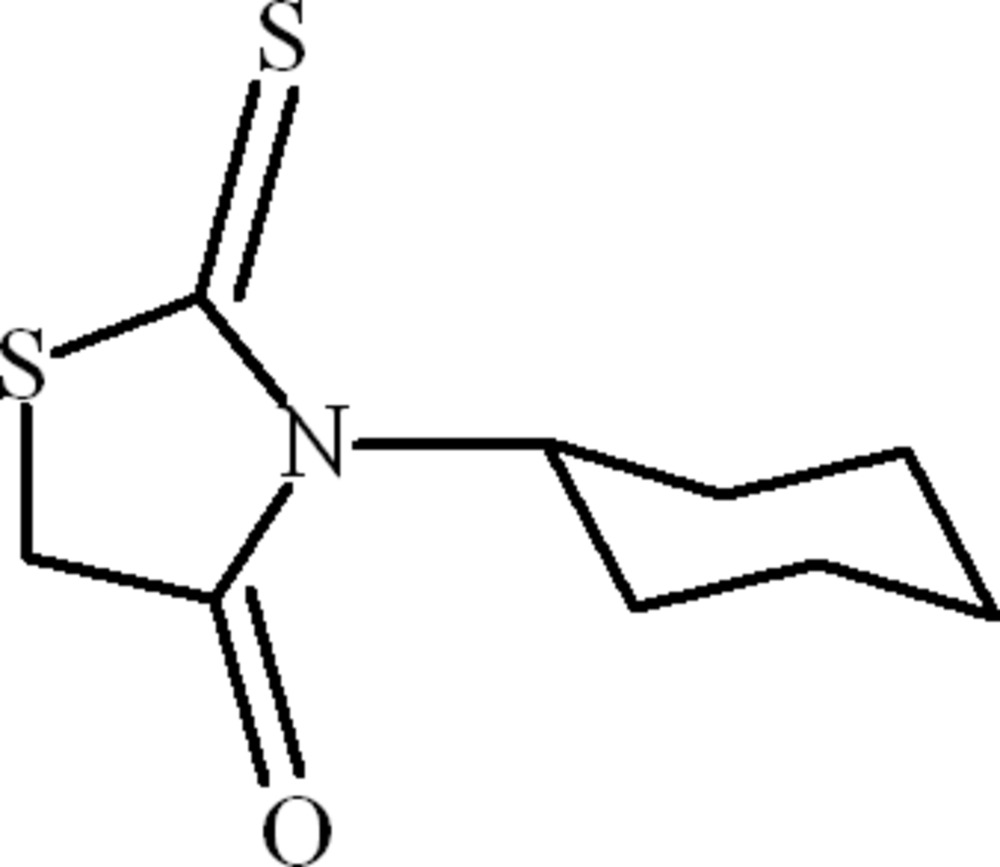


## Experimental

### 

#### Crystal data

C_9_H_13_NOS_2_
*M* *_r_* = 215.32
Monoclinic, 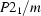

*a* = 7.3897 (3) Å
*b* = 7.0999 (4) Å
*c* = 10.3399 (5) Å
β = 107.535 (2)°
*V* = 517.29 (4) Å^3^
*Z* = 2
Mo *K*α radiation
μ = 0.48 mm^−1^
*T* = 296 K
0.36 × 0.25 × 0.23 mm

#### Data collection

Bruker Kappa APEXII CCD diffractometer
Absorption correction: multi-scan (*SADABS*; Bruker, 2005 ▶) *T*_min_ = 0.849, *T*_max_ = 0.897
5969 measured reflections
1390 independent reflections
1194 reflections with *I* > 2σ(*I*)
*R*_int_ = 0.028

#### Refinement

*R*[*F* ^2^ > 2σ(*F* ^2^)] = 0.034
*wR*(*F* ^2^) = 0.096
*S* = 1.07
1390 reflections
76 parameters
H atoms treated by a mixture of independent and constrained refinement
Δρ_max_ = 0.43 e Å^−3^
Δρ_min_ = −0.23 e Å^−3^

### 

Data collection: *APEX2* (Bruker, 2007 ▶); cell refinement: *SAINT* (Bruker, 2007 ▶); data reduction: *SAINT*; program(s) used to solve structure: *SHELXS97* (Sheldrick, 2008 ▶); program(s) used to refine structure: *SHELXL97* (Sheldrick, 2008 ▶); molecular graphics: *ORTEP-3* (Farrugia, 1997 ▶) and *PLATON* (Spek, 2009 ▶); software used to prepare material for publication: *WinGX* (Farrugia, 1999 ▶) and *PLATON*.

## Supplementary Material

Crystal structure: contains datablocks global, I. DOI: 10.1107/S1600536809045851/hb5207sup1.cif

Structure factors: contains datablocks I. DOI: 10.1107/S1600536809045851/hb5207Isup2.hkl

Additional supplementary materials:  crystallographic information; 3D view; checkCIF report

## Figures and Tables

**Table 1:** Hydrogen-bond geometry (Å, °)

*D*—H⋯*A*	*D*—H	H⋯*A*	*D*⋯*A*	*D*—H⋯*A*
C4—H4⋯S2	0.98	2.61	3.158 (2)	115
C5—H51⋯O1	0.97	2.51	3.095 (2)	119

**Fig. 1.**
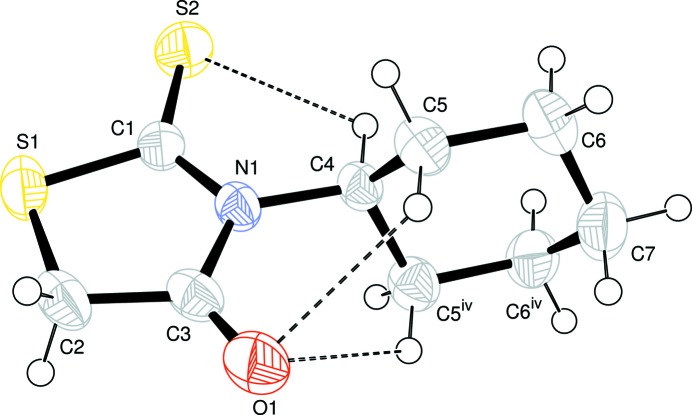
View of (I) with displacement ellipsoids drawn at the 50% probability level. The dotted lines represen the intramolecular H-bonds.

## supplementary crystallographic information

### Comment

Our group is involved in synthesizing various rhodanine derivatives for beta-lactamase and xanthine oxidase enzyme inhibition studies. In this context, we have already reported the preparation and crystal structures of (II) (5*Z*)-5-(2-Hydroxybenzylidene)-3-phenyl-2-thioxo-1,3- thiazolidin-4-one (Shahwar *et al.*, 2009*a*), (III) (5*E*)-5-(4-Hydroxy-3-methoxybenzylidene)-2-thioxo-1, 3-thiazolidin-4-one methanol monosolvate (Shahwar *et al.*, 2009*b*), (IV) (5*Z*)-5-(2-Hydroxybenzylidene)-2-thioxo-1,3-thiazolidin-4-one methanol hemisolvate (Shahwar *et al.*, 2009*c*) and (V) 3-(2-Methylphenyl)-2-thioxo-1,3-thiazolidin-4-one (Shahwar *et al.*, 2009d). The title compound (I, Fig. 1) is in continuation of synthesizing rhodanine derivatives for biological studies.

In (I), the rhodanine group A (N1/C1/S1/C1/C3/O1/S2) and the basal plane B (C5/C6/C5^i^/C6^i^; symmetry code: i = *x*, - *y* + 1/2, *z*) of cyclohexyl are planar and are perpendicularly oriented. The monomeric molecules are stabilized through intramolecular H-bondings (Table 1, Fig. 1) forming a S(5) and two S(6) ring motifs (Bernstein *et al.*, 1995). The apical C-atoms C4 and C7 of cyclohexyl are at a distance of 0.6430 (28) and -0.6667 (36) Å respectively, from the basal plane. There exist π–π interactions at a distance of 3.8140 (5) Å between the centroids of the heterocyclic rings.

### Experimental

The title compound was prepared by a three step reaction procedure. In the first step cyclohexylamine (9.9 g, 0.1 mol) and triethylamine (50.5 g, 0.5 mol) were stirred in ethanol (20 ml) followed by dropwise addition of CS_2_ (15.2 g, 0.2 mol) while keeping the flask in an ice bath. The precipitate obtained were filtered off and washed with diethyl ether.

In second step, a solution of sodium chloroacetate (11.6 g, 0.1 mol) and chloroacetic acid (18.9 g, 0.2 mol) was prepared in 50 ml distilled water. To this solution the precipitates obtained in first step were added gradually and stirred at 273 K. This mixture was stirred untill it turned clear yellow.

In third step the yellow mixture was mixed in 140 ml hot (363–368 K) hydrochloric acid (6 N) and stirred for five minutes to obtain colorless crystalline precipitates. These precipitates were recrystalized in chloroform to get colourless prisms of (I).

### Refinement

The coordinates of H2 were refined. The other H-atoms were positioned geometrically (C–H = 0.97–0.98 Å) and refined as riding with *U*_iso_(H) = 1.2*U*_eq_(C).

### Crystal data

**Table:**

C_9_H_13_NOS_2_	*F*(000) = 228
*M**_r_* = 215.32	*D*_x_ = 1.382 Mg m^−^^3^
Monoclinic, *P*2_1_/*m*	Mo *K*α radiation, λ = 0.71073 Å
Hall symbol: -P 2yb	Cell parameters from 1390 reflections
*a* = 7.3897 (3) Å	θ = 2.9–28.4°
*b* = 7.0999 (4) Å	µ = 0.48 mm^−^^1^
*c* = 10.3399 (5) Å	*T* = 296 K
β = 107.535 (2)°	Prism, colourless
*V* = 517.29 (4) Å^3^	0.36 × 0.25 × 0.23 mm
*Z* = 2	

### Data collection

**Table:**

Bruker Kappa APEXII CCD diffractometer	1390 independent reflections
Radiation source: fine-focus sealed tube	1194 reflections with *I* > 2σ(*I*)
graphite	*R*_int_ = 0.028
Detector resolution: 7.40 pixels mm^-1^	θ_max_ = 28.4°, θ_min_ = 2.9°
ω scans	*h* = −9→9
Absorption correction: multi-scan (*SADABS*; Bruker, 2005)	*k* = −9→9
*T*_min_ = 0.849, *T*_max_ = 0.897	*l* = −13→12
5969 measured reflections	

### Refinement

**Table:**

Refinement on *F*^2^	Primary atom site location: structure-invariant direct methods
Least-squares matrix: full	Secondary atom site location: difference Fourier map
*R*[*F*^2^ > 2σ(*F*^2^)] = 0.034	Hydrogen site location: inferred from neighbouring sites
*wR*(*F*^2^) = 0.096	H atoms treated by a mixture of independent and constrained refinement
*S* = 1.07	*w* = 1/[σ^2^(*F*_o_^2^) + (0.0474*P*)^2^ + 0.1418*P*] where *P* = (*F*_o_^2^ + 2*F*_c_^2^)/3
1390 reflections	(Δ/σ)_max_ < 0.001
76 parameters	Δρ_max_ = 0.43 e Å^−^^3^
0 restraints	Δρ_min_ = −0.23 e Å^−^^3^

### Special details

**Table:**

Geometry. Bond distances, angles *etc*. have been calculated using the rounded fractional coordinates. All su's are estimated from the variances of the (full) variance-covariance matrix. The cell e.s.d.'s are taken into account in the estimation of distances, angles and torsion angles
Refinement. Refinement of *F*^2^ against ALL reflections. The weighted *R*-factor *wR* and goodness of fit *S* are based on *F*^2^, conventional *R*-factors *R* are based on *F*, with *F* set to zero for negative *F*^2^. The threshold expression of *F*^2^ > σ(*F*^2^) is used only for calculating *R*-factors(gt) *etc*. and is not relevant to the choice of reflections for refinement. *R*-factors based on *F*^2^ are statistically about twice as large as those based on *F*, and *R*- factors based on ALL data will be even larger.

### Fractional atomic coordinates and isotropic or equivalent isotropic displacement parameters (Å^2^)

**Table:**

	*x*	*y*	*z*	*U*_iso_*/*U*_eq_	
S1	0.01379 (9)	0.25000	0.58014 (6)	0.0511 (2)	
S2	0.41214 (8)	0.25000	0.58341 (6)	0.0543 (2)	
O1	−0.1794 (2)	0.25000	0.19333 (18)	0.0591 (6)	
N1	0.1088 (2)	0.25000	0.35969 (16)	0.0351 (4)	
C1	0.1866 (3)	0.25000	0.4967 (2)	0.0363 (5)	
C2	−0.1778 (3)	0.25000	0.4253 (3)	0.0477 (7)	
C3	−0.0908 (3)	0.25000	0.3109 (2)	0.0412 (6)	
C4	0.2266 (2)	0.25000	0.26546 (19)	0.0356 (5)	
C5	0.1971 (2)	0.0715 (2)	0.18098 (16)	0.0450 (4)	
C6	0.3246 (2)	0.0739 (3)	0.08896 (17)	0.0527 (5)	
C7	0.2925 (4)	0.25000	0.0025 (3)	0.0590 (8)	
H2	−0.255 (2)	0.142 (3)	0.4183 (17)	0.0573*	
H4	0.35945	0.25000	0.32201	0.0427*	
H51	0.06529	0.06226	0.12627	0.0540*	
H52	0.22695	−0.03748	0.24022	0.0540*	
H61	0.45638	0.06820	0.14408	0.0633*	
H62	0.29826	−0.03620	0.03070	0.0633*	
H71	0.16366	0.25000	−0.05835	0.0707*	
H72	0.37843	0.25000	−0.05230	0.0707*	

### Atomic displacement parameters (Å^2^)

**Table:**

	*U*^11^	*U*^22^	*U*^33^	*U*^12^	*U*^13^	*U*^23^
S1	0.0636 (4)	0.0480 (3)	0.0539 (3)	0.0000	0.0361 (3)	0.0000
S2	0.0461 (3)	0.0671 (4)	0.0438 (3)	0.0000	0.0046 (2)	0.0000
O1	0.0307 (7)	0.0850 (13)	0.0582 (10)	0.0000	0.0084 (7)	0.0000
N1	0.0300 (7)	0.0394 (8)	0.0382 (8)	0.0000	0.0138 (6)	0.0000
C1	0.0431 (9)	0.0288 (8)	0.0401 (10)	0.0000	0.0173 (8)	0.0000
C2	0.0417 (10)	0.0390 (11)	0.0722 (15)	0.0000	0.0318 (10)	0.0000
C3	0.0303 (8)	0.0395 (10)	0.0554 (12)	0.0000	0.0153 (8)	0.0000
C4	0.0273 (7)	0.0445 (10)	0.0365 (9)	0.0000	0.0121 (7)	0.0000
C5	0.0444 (7)	0.0422 (8)	0.0528 (8)	0.0012 (6)	0.0213 (6)	−0.0023 (6)
C6	0.0485 (8)	0.0602 (10)	0.0554 (9)	0.0028 (7)	0.0246 (7)	−0.0120 (8)
C7	0.0561 (13)	0.0816 (18)	0.0456 (12)	0.0000	0.0250 (10)	0.0000

### Geometric parameters (Å, °)

**Table:**

S1—C1	1.743 (2)	C6—C7	1.514 (3)
S1—C2	1.789 (3)	C2—H2	0.95 (2)
S2—C1	1.637 (2)	C2—H2^i^	0.95 (2)
O1—C3	1.195 (3)	C4—H4	0.9800
N1—C1	1.359 (3)	C5—H51	0.9700
N1—C3	1.408 (3)	C5—H52	0.9700
N1—C4	1.489 (2)	C6—H61	0.9700
C2—C3	1.507 (3)	C6—H62	0.9700
C4—C5	1.5172 (18)	C7—H71	0.9700
C4—C5^i^	1.5172 (18)	C7—H72	0.9700
C5—C6	1.528 (2)		
			
C1—S1—C2	93.28 (11)	C3—C2—H2^i^	109.3 (10)
C1—N1—C3	116.24 (17)	H2—C2—H2^i^	108.4 (16)
C1—N1—C4	122.34 (16)	N1—C4—H4	107.00
C3—N1—C4	121.43 (15)	C5—C4—H4	107.00
S1—C1—S2	120.37 (12)	C5^i^—C4—H4	107.00
S1—C1—N1	111.90 (16)	C4—C5—H51	110.00
S2—C1—N1	127.73 (17)	C4—C5—H52	110.00
S1—C2—C3	107.02 (16)	C6—C5—H51	110.00
O1—C3—N1	124.0 (2)	C6—C5—H52	110.00
O1—C3—C2	124.5 (2)	H51—C5—H52	108.00
N1—C3—C2	111.56 (18)	C5—C6—H61	109.00
N1—C4—C5	111.45 (9)	C5—C6—H62	109.00
N1—C4—C5^i^	111.45 (9)	C7—C6—H61	109.00
C5—C4—C5^i^	113.30 (14)	C7—C6—H62	109.00
C4—C5—C6	109.92 (13)	H61—C6—H62	108.00
C5—C6—C7	111.15 (17)	C6—C7—H71	109.00
C6—C7—C6^i^	111.4 (2)	C6—C7—H72	109.00
S1—C2—H2	111.4 (11)	H71—C7—H72	108.00
S1—C2—H2^i^	111.4 (11)	C6^i^—C7—H71	109.00
C3—C2—H2	109.3 (10)	C6^i^—C7—H72	109.00
			
C2—S1—C1—S2	180.00 (1)	C4—N1—C3—C2	180.00 (1)
C2—S1—C1—N1	0.00 (1)	C1—N1—C4—C5	116.17 (11)
C1—S1—C2—C3	0.00 (1)	C3—N1—C4—C5	−63.83 (11)
C3—N1—C1—S1	0.00 (1)	S1—C2—C3—O1	180.00 (1)
C3—N1—C1—S2	180.00 (1)	S1—C2—C3—N1	0.00 (1)
C4—N1—C1—S1	180.00 (1)	N1—C4—C5—C6	−178.24 (13)
C4—N1—C1—S2	0.00 (1)	C5^i^—C4—C5—C6	55.10 (17)
C1—N1—C3—O1	180.00 (1)	C4—C5—C6—C7	−54.99 (19)
C1—N1—C3—C2	0.00 (1)	C5—C6—C7—C6^i^	56.9 (2)
C4—N1—C3—O1	0.00 (1)		

Symmetry codes: (i) *x*, −*y*+1/2, *z*.

### Hydrogen-bond geometry (Å, °)

**Table:**

*D*—H···*A*	*D*—H	H···*A*	*D*···*A*	*D*—H···*A*
C4—H4···S2	0.98	2.61	3.158 (2)	115
C5—H51···O1	0.97	2.51	3.095 (2)	119
